# Hallmarks of ageing in human skeletal muscle and implications for understanding the pathophysiology of sarcopenia in women and men

**DOI:** 10.1042/CS20230319

**Published:** 2023-11-21

**Authors:** Antoneta Granic, Karen Suetterlin, Tea Shavlakadze, Miranda D. Grounds, Avan A. Sayer

**Affiliations:** 1 AGE Research Group, Translational and Clinical Research Institute, Faculty of Medical Sciences, Newcastle University, U.K.; 2 NIHR Newcastle Biomedical Research Centre, Newcastle University and Newcastle Upon Tyne Hospitals NHS Foundation Trust, Newcastle Upon Tyne, U.K.; 3 John Walton Muscular Dystrophy Research Centre, Institute of Genetic Medicine, Newcastle University, Centre for Life, Newcastle upon Tyne, U.K.; 4 Regeneron Pharmaceuticals Inc., Tarrytown, New York, NY, U.S.A.; 5 Department of Anatomy, Physiology and Human Biology, School of Human Sciences, the University of Western Australia, Perth, WA 6009, Australia

**Keywords:** hallmarks of ageing, sarcopenia, skeletal muscle

## Abstract

Ageing is a complex biological process associated with increased morbidity and mortality. Nine classic, interdependent hallmarks of ageing have been proposed involving genetic and biochemical pathways that collectively influence ageing trajectories and susceptibility to pathology in humans. Ageing skeletal muscle undergoes profound morphological and physiological changes associated with loss of strength, mass, and function, a condition known as sarcopenia. The aetiology of sarcopenia is complex and whilst research in this area is growing rapidly, there is a relative paucity of human studies, particularly in older women. Here, we evaluate how the nine classic hallmarks of ageing: *genomic instability, telomere attrition, epigenetic alterations, loss of proteostasis, deregulated nutrient sensing, mitochondrial dysfunction, cellular senescence, stem cell exhaustion*, and *altered intercellular communication* contribute to skeletal muscle ageing and the pathophysiology of sarcopenia. We also highlight five novel hallmarks of particular significance to skeletal muscle ageing: *inflammation, neural dysfunction, extracellular matrix dysfunction, reduced vascular perfusion*, and *ionic dyshomeostasis*, and discuss how the classic and novel hallmarks are interconnected. Their clinical relevance and translational potential are also considered.

## Introduction

Hallmarks have been traditionally used as organising principles to identify distinctive characteristics or underlying biological causes of a process such as ageing in many human tissues and animal models [1–3] and disease [4,5]. Nine unifying hallmarks of biological ageing, termed primary, antagonistic, or integrative hallmarks, have been proposed involving genetic and biochemical pathways that collectively influence ageing trajectories and susceptibility to pathology in humans [1]. These are *genomic instability, telomere attrition, epigenetic alteration, loss of proteostasis* (primary), *deregulated nutrient sensing, mitochondrial dysfunction, cellular senescence* (antagonistic), *stem cell exhaustion*, and *altered intercellular communication* (integrative). Recent addition of new hallmarks such as *compromised autophagy, chronic inflammation*, and *dysbiosis* [2,3], *altered mechanical properties* and *splicing dysregulation* [3,6] emphasises the complexity of the ageing process. As originally proposed, experimental manipulation of these hallmarks may either accelerate or ameliorate mammalian ageing [1,2]. Hallmarks have been used as a contextual framework to classify the multiplicity of mechanisms governing normal ageing in tissues and body systems [7,8] and they have been mapped to age-related diseases [4,9].

This review investigates the relevance of the hallmarks of ageing to the specific situation of ageing skeletal muscle and sarcopenia, a condition characterised by progressive and generalised loss of skeletal muscle strength, mass, and function [10,11]. Here we give a broad overview of the main mechanisms that are likely to drive the pathophysiology of sarcopenia in normal skeletal muscle of ageing women and men in the context of these hallmarks. This focus on human rather than animal skeletal muscle biology also allows consideration of the clinical relevance and translational potential of hallmarks for sarcopenia.

Specifically, this review addresses the following four objectives to:

1. Evaluate the nine classic hallmarks of ageing and highlight five novel hallmarks potentially most relevant for ageing skeletal muscle and sarcopenia in humans
2. Highlight the existence of large literature involving animal studies and a relative paucity of human studies particularly in older women
3. Emphasise the interconnectedness of hallmarks and need for interdisciplinary approach to integrate biology of ageing muscle
4. Identify clinical relevance and translational potential of hallmarks for sarcopenia.

The review has three main parts. The first and second parts (**Human skeletal muscle and ageing** and **Hallmarks of ageing in skeletal muscle and sarcopenia)** address objectives 1 and 2, and the third part (**Clinical relevance and translational potential**) discusses objectives 3 and 4, followed by **Conclusions**.

### Human skeletal muscle and ageing

Maintaining skeletal muscle health in terms of muscle strength, mass, and function (physical performance) [12], is a prerequisite to increase the healthspan [13,14]. Skeletal muscle comprises approximately 40% of total human body mass and is a complex tissue [15] that serves multiple functions, from main mechanistic contraction for movement, to thermogenesis and metabolism [16].

**Figure 1:**
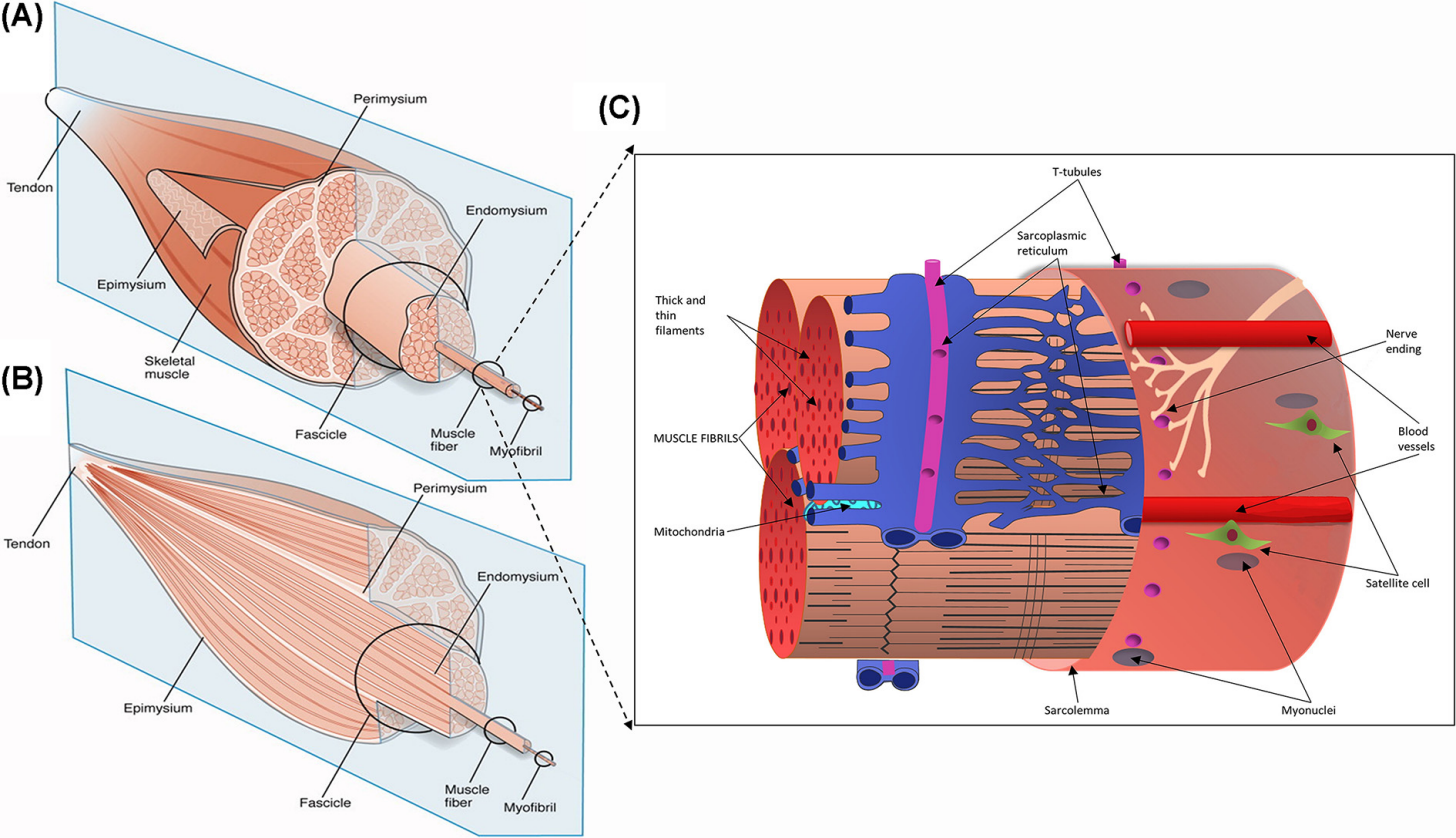
Schematic diagram of skeletal muscle tissue and of a single muscle fibre organisation Panels (**A,B**) present schematic diagrams of the skeletal muscle tissue and muscle extracellular matrix (ECM)-tendon organisation. The muscle ECM is categorised into epimysium (a layer surrounding the entire muscle), perimysium (a layer surrounding muscle fascicles or the muscle fibre bundles, which are further grouped together to form the muscle tissue), and endomysium (a basal lamina that is in intimate contact with individual myofibres). In the longitudinal section of the muscle (panel **B**), the endomysium is enclosed within the fascicles, whereas the perimysium is presented continuously with the tendon. Panel (**C**) represents a single myofibre comprised of the myofibrils organised into sarcomeres enclosed in the sarcolemma. The sarcoplasmic reticulum entangles the fibrils, and the transverse (T) tubules intersect them. Mitochondria are secured near the T-tubules to sarcoplasmic reticulum junction and (or) are found in subsarcolemmal regions. Along the length of the myofibres are nerves (attached at the NMJ) and capillaries (often near satellite cells). Panels **A** and **B** from Gillies, A.R. and Lieber, R.L. (2011) Structure and function of the skeletal muscle extracellular matrix. *Muscle Nerve* **44**, 318–331. Panel **C** was adapted from [15] and used with the permission of Wiley.

Skeletal muscle tissue consists of long contractile multinucleated myofibres filled with specialised contractile proteins organised into sarcomeres, with invaginations of the plasma membrane (sarcolemma), known as T-tubules to facilitate rapid and coordinated conversion of excitation into contraction, and mitochondria tethered at sites of high energy need. Each myofibre is connected to a nerve via the neuromuscular junction (NMJ) to stimulate myofibre contraction and there is a rich vascular supply with a network of capillaries around the myofibres (Figure 1). Specialised extracellular matrix (ECM) rich in laminins, known as the basal lamina (or basement membrane) is in intimate contact with the sarcolemma of each myofibre. In mature muscles, mononucleated muscle precursor cells called satellite cells are located on the myofibre surface between the sarcolemma and basal lamina [16,17] (Figure 1): these cells are responsible for muscle regeneration following necrosis resulting from injury. Other mononucleated interstitial cells include fibroblasts, adipocytes, and mesenchymal stem cells, surrounded by complex ECM molecules.

**Figure 2:**
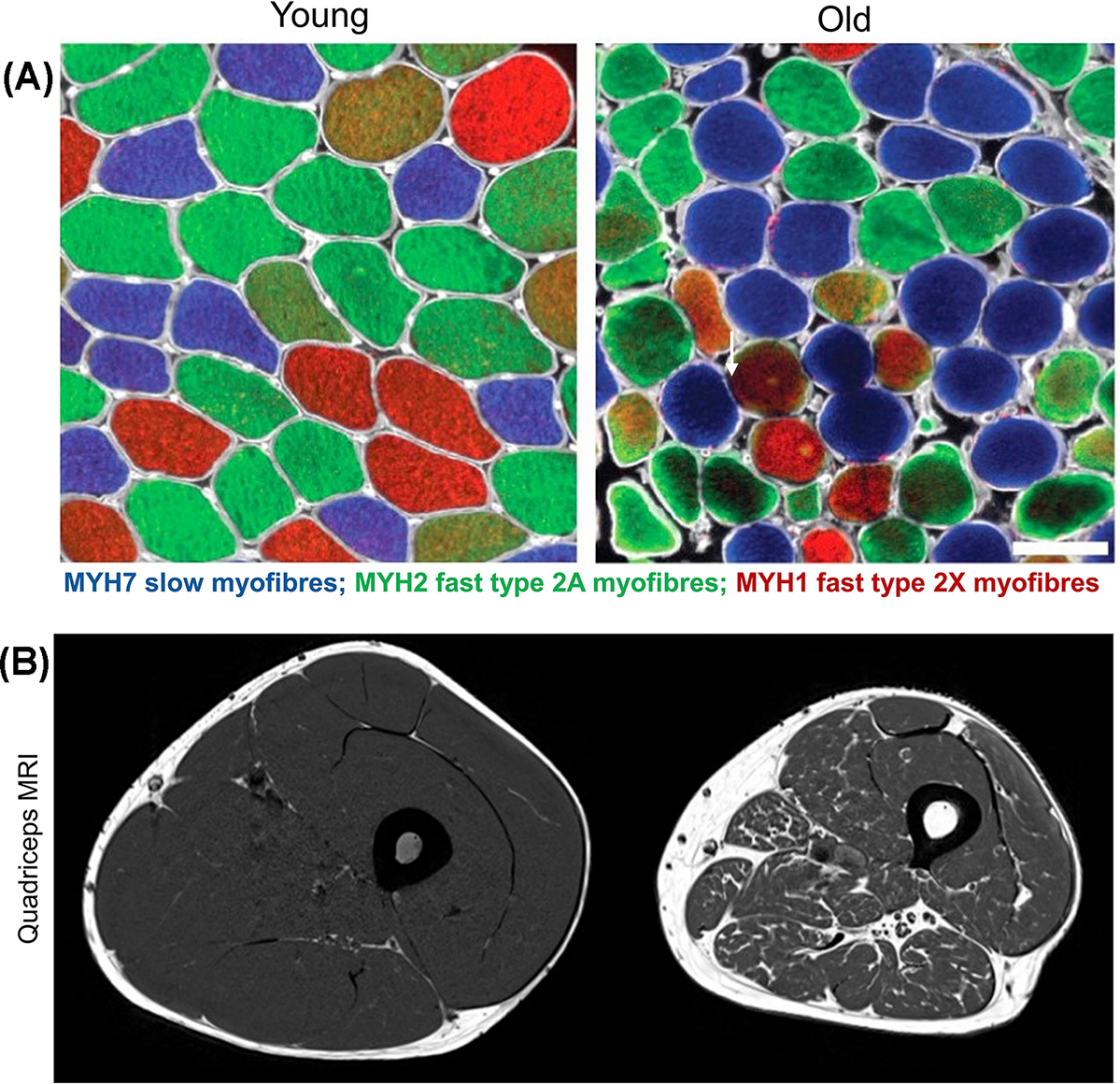
Comparison of skeletal muscle morphology and architecture in young and old adults Panel (**A**) depicts representative cross-sections of the *vastus lateralis* (*VL*) muscle biopsies from young (left; aged ∼24 years) and old (right; aged ∼70 years) healthy, active men, immuno-stained with antibodies specific to adult myosin heavy chain (MYH) isoforms: blue (anti-MYH7, slow type 1 myofibres), green (anti-MYH2, fast type 2A myofibres), and red (anti-MYH1, fast type 2X myofibres) (scale bar: 100 μm). Compared with the relatively uniform size of myofibres in young muscles (left; Panel **A**), the main features of old muscle are a wider range (variability) of size and shapes especially in type 2 myofibres (right; Panel (**A**), white arrow). Panel (**B**) shows a magnetic resonance imaging (MRI) of human thigh with area of quadriceps muscle (grey) and surrounding fatty tissue (white) in young (left) compared with loss of muscle mass in older men (right). Panel (**A**) from [38] was used with the permission from Cell Press. Panel (**B**) from Herrmann, M., Engelke, K., Ebert, R., Müller-Deubert, S., Rudert, M., Ziouti, F. et al. (2020) Interactions between Muscle and Bone-Where Physics Meets Biology. *Biomolecules***10**, 432, with permission from MDPI.

Most adult human muscles are a mixture of slow-twitch type 1 (oxidative) and fast-twitch type 2 myofibres (glycolytic) [18] (Figure 2**A**), with the myofibre type being primarily defined by a motor nerve (motorneuron) activity [19]. Slow and fast myofibres differ by their contractile, biochemical, and metabolic phenotypes, contributing to the heterogeneity of over 600 muscles in the human body that differ in their architecture and function [16,18–25].

Age-related decrease in muscle mass and strength starts around the fourth decade of life [26–28], and accelerates in later life [26,29]. Estimates from longitudinal studies of adults (aged ≥75 years) show annual loss in muscle mass of 0.64–0.70% in men and 0.80–0.98% in women, with an even steeper decline in muscle strength of 3–4% in men and 2.5–3% women [29]. Skeletal muscle is malleable organ responsive to intrinsic and extrinsic stimuli such as exercise, diet, and inflammation across the lifecourse [22,26–29]: sarcopenia is characterised by a decline in muscle mass and function beyond the normal clinically defined cut-offs [10,11,28]. Magnetic resonance imaging (MRI) of old human thighs (*vastus lateralis, VL*) shows decreased lean muscle mass and replacement of muscle tissue by fat (Figure 2**B**), which accounts for deterioration of muscle quality and function in sarcopenia. However, the age-related changes are not uniform across all muscles and the extent of sarcopenia varies between muscles in different locations with different function [25].

At the cellular level, some key features of skeletal muscle ageing are increased myofibre variability and decreased myofibre cross-sectional area (atrophy), as demonstrated by analyses of biopsied muscle tissue sections (Figure 2**A**); also net loss of myofibre numbers [23,30,31] (reviewed in [32–35]) and functional denervation of myofibres caused by destabilisation of NMJs [23,36]. To this end, fast type myofibres are more affected compared with slow, a phenomenon known as preferential atrophy and loss of type 2 myofibres [35]; many of these fast myofibres become denervated and are re-innervated by slow motorneurons, so they survive but lose their original identity [22–25,30–38]. We consider that loss of motor-innervation is a hallmark of skeletal muscle ageing [37] (see *Hallmark: Neural dysfunction*). Additionally, single-myofibre proteomics reveal metabolic changes in fast, but not slow, myofibres in old versus young men [38]) (see *Hallmark: Deregulated nutrient sensing*).

**Figure 3:**
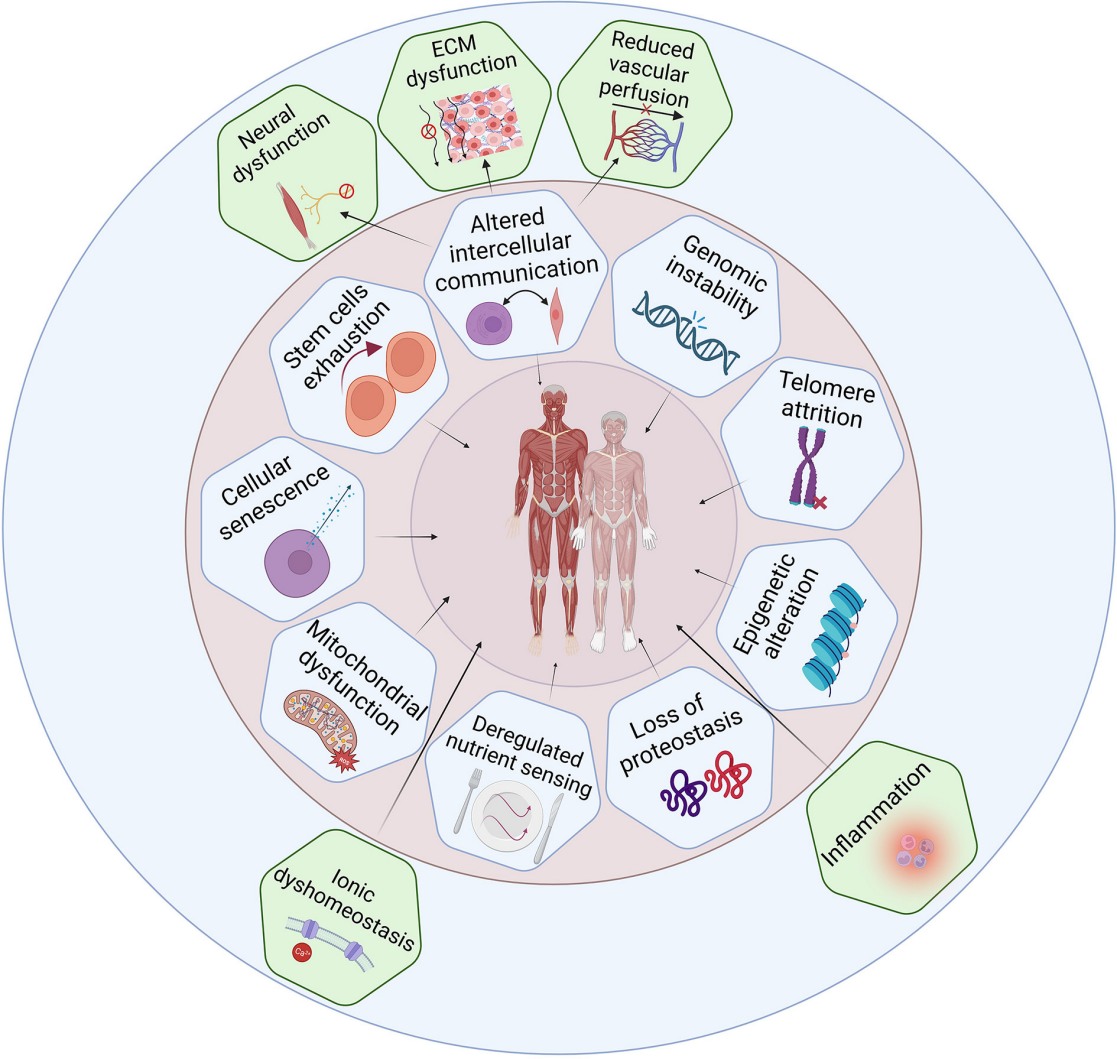
Hallmarks of ageing as applied to human skeletal muscle Hallmarks of ageing were evaluated for their involvement in skeletal muscle ageing and sarcopenia. The nine classic hallmarks [1] are depicted in the inner (pink) circle. The outer (blue) circle highlights potential new hallmarks of skeletal muscle ageing and sarcopenia. Created with BioRender.com.

The mechanisms underlying sarcopenia in ageing human muscles are complex [14,39–41] and the interconnectedness of cellular components and body systems, sex-specific differences, and cross-species comparison makes understanding the predominant driving mechanisms challenging [42,43]. Here we evaluate the relevance to ageing skeletal muscle and sarcopenia for each of the classic nine hallmarks of ageing [1] and suggest five novel skeletal muscle-specific hallmarks (Figure 3).

## Hallmarks of ageing in skeletal muscle and sarcopenia

### Genomic instability

The accumulation of genetic damage caused by endogenous and environmental factors is a key feature of ageing and age-related disease [44,45] inversely associated with the lifespan in mammals [46]. This manifests in various forms, including somatic mutations (e.g., point mutations, translocations and deletions occurring in nuclear and mitochondrial (mt) DNA), telomere shortening, and chromosomal aneuploidy. Loss of heterochromatin, resulting in de-repression and activation of retrotransposon activity is another consequence of ageing potentially leading to somatic mutations [47,48]. DNA damage that escapes the DNA damage detection or subsequent illigitimate repair -the machinery of which also becomes error-prone with age [49] -and activation of repressed genome elements, may cause detrimental changes in gene transcription and translation, resulting in dysfunctional cells that, if not cleared, contribute to dyshomeostasis of tissues and organs. Here, we briefly discuss the relevance of nuclear and mtDNA alterations and activation of retrotransposable elements (RTEs) in human skeletal muscle with ageing.

Threre are many factors that contribute to DNA damage in old tissues, including skeletal muscle, but the direct correlation to ageing is not fully elucidated (reviewed in [50]). Reactive oxygen species (ROS) such as free radicals are regarded as a major cause of damage to DNA (nuclear and mitochondrial) and also proteins, lipids and other cell components (reviewed in [51]) in skeletal muscles. ROS are naturally produced during muscle metabolism, contraction, regeneration, inflammation, and hypoxia [50,52]. Damaged DNA can contribute to muscle ageing by altering gene expression and inducing cellular senescence (see *Hallmark: Cellular senescence*). Evidence for other types of DNA damage, however, in ageing and sarcopenic skeletal muscle is limited. In an early study of 66 adults aged 25–93 years (47% women) 8-hydroxy-2-deoxyguanosine, a marker of oxidative DNA damage was substantially increased in *VL* and *vastus medialis* muscle with age [53]. Oxidatively damaged DNA can block transcription, decrease protein synthesis and degradation, and attenuate synthesis of key muscle proteins (e.g., actin and myosin), consequently decreasing muscle strength and function (reviewed in [50,54]).

It is well established that chemotherapy and radiation cause excessive DNA damage in cancer and normal cells triggering mutagenesis, senescence, and apoptosis (reviewed in [55]). Chemotherapy may further contribute to DNA oxidation and damage by increasing ROS and oxidative stress in ageing muscle [56]. Studies with middle aged and older cancer patients undergoing antineoplastic treatments for various cancers have observed exacerbated loss of muscle mass (cancer cachexia) and increased risk of sarcopenia (loss of muscle mass and function) by not fully understood mechanisms (reviewed in [56,57]). However, reports also show muscle gain or no change in muscle mass in subpopulation of cancer patients with stable clinical course of the disease [56,58], including older patients [58]. Taken together, these examples indicate a complex relationship between DNA damaging oncologic agents, cancer cachexia and sarcopenia in cancer patients.

Mitochondrial DNA represents 0.93% of total DNA in human skeletal muscle (reviewed in [59]). An increase in deletions and point mutations in mtDNA in post-mitotic tissues such as skeletal muscle has been observed during normal ageing in studies with bulk muscle tissue (reviewed in [60]) [61,62] and in myofibres [63]. Histochemical analyses of 12 *VL* muscle biopsies from men and women aged 49–92 years, showed that older muscles have higher number of myofibres with electron transport system (ETS) enzymatic abnormalities and respiratory chain deficiency (from 6% to 31%) [63]. Notably, all myofibres with the ETS abnormalities were harbouring mtDNA-deletion mutations as shown by polymerase-chain reaction (PCR) [63]. Another study used high-throughput droplet digital PCR for quantification of mtDNA deletion frequency in 14 *VL* muscle biopsies from men aged 20–80 years [61] and found a 98-fold increase of these deletions between the youngest and the oldest age [61]. A follow-up study by the same group showed an 18-fold increase (from 0.008 to 0.15%; 10.4%/year) in deletions from ages 50–86 years in men and women, which correlated with a lower physical performance [62].

Another ultra-sensitive high-throughput mtDNA deletion detection method called LostArc [59] was developed to assess the full deletion spectrum across the mtDNA genome of myofibres in *VL* muscle in individuals aged 17-93 years. This showed that, while mtDNA levels and copy numbers are not affected by age, deletion rates increase with age favouring genes that support electron transport and the oxidative phosphorylation (OXPHOS) in older adults and sparing those involved in mtDNA replication [59]. However, mtDNA ablations (the deleted mtDNA fraction) varied by age and increased linearly after age 60, possibly contributing to mitochondrial dysfunction in older muscles [59].

Taken together, destabilisation of the mitochondrial genome observed in ageing skeletal muscle may lead to dysfunctional ETS, focal respiratory chain deficiency, decline in energy production and myofibre atrophy [64] and thus contribute to sarcopenia. Decline in mitochondrial function as a hallmark of skeletal muscle ageing is discussed further in *Hallmark: Mitochondrial dysfunction*.

Increasing evidence suggets that transposons (retrotransposable elements, RTEs), genetic sequences that can move within the genome, play a role in ageing and age-related diseases in eukaryotes [47,65,66]. The human genome comprises 35% of RTEs; the activation of a small sub-set of those that are functionally active in adult human somatic tissue appears to be harmful and may promote age-related phenotypes [66–68]. RTEs act by triggering DNA damage, mutagenesis, aberrant transcription, alternative splicing, genomic instability, innate immune response, and inflammation [47,69–71]. To counteract their activity, organisms have developed several mechanisms for silencing active RTEs such as DNA methylation, histone modification, and RNA silencing pathways [65,70,72].

The only active RTE in the human genome are LINE1 (long-interspersed element), which comprises 17% of the human genome [65]. Activation of LINE1 has been shown in senescent human fibroblasts *in vitro* [48], and old rodent tissues, including skeletal muscle [73,74]. Studies that describe activity of RTEs in human muscle ageing are limited (reviewed in [75]). One human study showed de-repression of LINE1 and the resulting increase in LINE1 mRNA *VL* muscle biopsies in 13 older men and women aged (58 ± 8 years), compared with 15 younger participants (aged 23 ± 3 years) [76]. In the context of ageing, increased LINE1 mRNA expression results in cytoplasmic accumulation (rather than retrotrasposition) of LINE1 cDNA and this, in turn, induces type-1 interferon (IFN-I) response and activation of pro-inflammatory pathways (inflammaging) in mice [73]. To this end, increased interferon-responsive genes and inflammation are a shared feature of ageing skeletal muscles, as well as other tissues, in rodents [77,78] and with some markers also observed in humans [6]. One study in humans proposed that LINE1 expression in skeletal muscles may be suppressed by exercise [75] but these initial findings require further confirmation.

In summary, emerging evidence from human studies indicates that oxidative damage to nuclear DNA, mtDNA alterations and transposons may play a role in muscle ageing and possibly sarcopenia by promoting mitochondrial dysfunction and inflammation, and cellular senescence.

### Telomere attrition

Telomere dynamics are proposed as a vital component of human ageing [79] characterised by a progressive shortening of telomeres (repetitive DNA sequences at the ends of chromosomes) in dividing cells, which triggers a DNA damage signal response and replicative senescence *in vitro* [79,80]. Telomere lengths (TLs) have been studied across human [81,82] and rodent tissues [83] and often in comparison with blood cells (leukocytes), which are used as a reference for TLs of the entire organism and a biomarker of ageing [84]. Although human blood samples are relatively easy to obtain to measure leukocyte TLs, compared with (invasive) muscle biopsies (to measure TLs in myofibres, satellite cells, or muscle-associated mononucleated cells), the leukocyte TLs cannot be considered a meaningful biological marker for a multidimensional age-related condition such as sarcopenia [85]. Also, there is a great heterogeneity in (leukocyte) TLs at the intracellular, intercellular, and individual level established using various techniques [86], with no standardised protocol and hight intra- and inter-laboratory technical variations [87].

Human studies of skeletal muscle telomere dynamics with ageing are less comprehensive and overall show relative TL stability in myonuclei with age [81,88,89], and malleability to environmental factors (e.g., longer telomeres being associated with higher levels of physical activity compared with a sedentary lifestyle) [90,91]. Inter-individual telomere variability in muscle as a minimally proliferative tissue appears to be established largely in early life during the normal growth phase [92,93], but this conclusion needs to consider which specific cells these data relate to (as listed above). For example, the TLs of satellite cells decrease during the first two decades of human life (a period of muscle growth), while TLs in myonuclei remains constant from birth to 86 years, indicating stability and very low turnover [88].

In the Genotype-Tissue Expression (GTEx) project, TL analyses of over 20 tissue types from 952 individuals aged 20–70 years revealed high inter-individual variability in TLs in most tissues with ageing, except in skeletal muscle. However, when leukocyte TLs were used as a reference of the entire body TL, they correlated moderately with TLs of most tissues (*r* range 0.15-0.37) [81]. A comparative study of telomere attrition in the minimally proliferative (fat, muscle) and maximally proliferative (skin, leukocytes) tissues from racially diverse samples of men and women aged 19–77 years, observed comparable age-dependent telomere attrition rates per year [92]. Although TLs were the longest in muscle and shortest in leukocytes, they still correlated between the tissues [92]. In another study involving young and old healthy men (aged 18–87 years), TLs in muscle and leukocytes were correlated across the lifecourse (*r* = 0.26) [94]. Individuals with longer leukocyte telomeres had longer muscle telomeres. Taken together, these studies infer early life origins of the TL differences across the tissues and suggest a shared endogenous mechanisms of TL regulation.

Currently, it is not known whether muscle telomere dynamics associate with muscle strength and mass during ageing, since sarcopenia has been mostly explored in relation to leukocyte TLs in human studies. The results from the studies using leukocyte TLs as a reference for myofibre TLs are inconsistent and warrant further investigation [84,95] to clarify whether skeletal muscle telomere attrition plays any role in muscle ageing and sarcopenia.

### Epigenetic alteration

Somatic cells are susceptible to epigenetic modifications, that change gene expression without modifying the base genetic code. DNA methylation and histone acetylation are the most studied epigenetic modifications, the former being well described in ageing skeletal muscle in bulk tissue [96–99]. Epigenetic changes are interconnected with other hallmarks of ageing and can lead to dysregulated nutrient sensing, mitochondrial dysfunction, and cellular senescence (discussed in [1,2]).

An altered epigenetic landscape in aged human skeletal muscle was first described by Zykovich et al. [96] using a genome-wide study of DNA methylation. DNA methylation arrays containing 450,000 CpG sites revealed hypermethylation across the genome in muscles from older men (aged 68–89 years) compared with younger men (aged 18–27 years). Over 5900 CpG sites were differentially methylated (dmCpG) between the groups, of which 92% were hypermethylated. The hypermethylation sites were under-represented in promoter regions and over-represented within the gene bodies (the central and 3′ regions), especially in genes that guide the formation of the NMJ. 500 CpG sites were able to distinguish between old and young muscle, creating the first epigenetic clock of muscle ageing.

A human muscle-specific epigenetic clock was then generated from 682 skeletal muscle samples and 12 independent methylome datasets (aged 18–89 years, 22% women, 99% white). This showed that the methylation status of 200 CpG sites can predict muscle chronological age [97]. Exploration of DNA methylation patterns revealed 180 differentially methylated regions (DMRs) with advancing age, equally balanced between hyper- and hypomethylation. A further update of the muscle epigenetic clock involved a large-scale epigenome-wide association study (EWAS) meta-analysis of skeletal muscle ageing from 10 studies and 908 muscle methylomes (men and women aged 19–89 years). The study identified over 6700 DMRs spanning >6300 unique genes involved in muscle structure development, contraction, and calcium transport regulation [98]. Global hypermethylation of human skeletal muscle with ageing and methylation patterns have been further confirmed in a large study involving 850,000 CpG sites in muscle tissue and isolated heterogenous muscle-derived human primary cells (HDMCs) from old (mean age 83 years) and young adults (mean age 27 years) [99]. Enriched hypermethylation in old muscle tissue was observed for various cellular pathways (e.g., mitogen-activated protein kinase [MAPK], phosphatidylinositol3-AKT-protein kinase B-mammalian target of rapamycin [PI3K-AKT-mTOR], and p53 signalling), axon-guidance, and Hippo-signalling pathway (proposed to control muscle mass and function [100]). Differential methylation analysis showed marked hypermethylation of the *HOX* genes (developmental regulatory and musculoskeletal patterning genes [101]) only in old human muscle tissue and cells [99].

The relationship between muscle methylome and sarcopenia have been investigated in a cohort of 83 older men (mean age: 76 years) from the Hertfordshire Sarcopenia Study (HSS) and HSS extension (14% with sarcopenia). The dmCpGs associated with sarcopenia were enriched in genes linked to myotube fusion (e.g., homophilic cell adhesion via plasma membrane adhesion molecules), oxidative phosphorylation, and voltage-gated calcium channels [102]. There was an overlap in the number of dmCpGs associated with sarcopenia and muscle mass, plus sarcopenia and muscle function (strength and gait speed) [102]. The results suggest that epigenetic alteration of specific genes may contribute to impaired muscle function in older men; yet to be confirmed in larger studies of older women [103,104].

Taken together, these results (mainly for older men), confirm DNA methylation of tissue-specific genes in skeletal muscle and their association with muscle characteristics, with flexibility of the muscle epigenetic landscape revealed by increasing detail in methylome sequencing and powerful EWAS meta-analyses and yet to be determined at single-cell resolution [105]. These data strongly support epigenetic alterations as a hallmark of ageing associated with sarcopenia; however, epigenetic studies of sarcopenia in older women are lacking.

### Loss of proteostasis

A balanced process of protein synthesis and degradation controls myofibre size, protein content and protein renewal. Extensive literature suggests a decline in protein homeostasis (or proteostasis) as a hallmark of ageing and age-associated disease [1,2,9,106–108]. Dysregulation of proteostasis leading to myofibre atrophy and accumulation of misfolded and aggregated proteins has been relatively well studied in ageing rodents, with limited studies in humans [6,109–111]. These studies suggest a very complex mis-regulation of many aspects of proteostasis in old muscles.

An extensive discovery study that compared the proteome from *VL* muscles of healthy men and women (aged 20–87 years), found that the abundance of ribosomal proteins and chaperones decreases with ageing [6]. Depletion of ribosomal proteins may result in lower protein synthesis, protein turnover, and, ultimately, a failure to replace damaged contractile machinery, while a decrease in chaperones may result in diminished chaperone-mediated autophagy [6].

One key pathway that controls protein homeostasis in skeletal muscle is the AKT-mTOR complex 1 (mTORC1) pathway, which is dysregulated in old muscle. As discussed below, age-related dysregulation of this pathway has been widely studied in rodents, while human data are limited. In healthy muscles, activation of AKT downstream of anabolic factor receptors, such as insulin-like growth factor 1 (IGF-1), stimulates the mTORC1-dependent protein synthesis [112]. Paradoxically, at least in rodents, mTORC1 is hyperactivated in old muscles [113–116]. Such an increase in mTORC1 signalling is associated with progression of sarcopenia in rats and occurs in parallel with elevated markers of muscle protein ubiquitination and suppressed markers of autophagy [113–116]. To this end, therapeutic inhibition of mTORC1 signalling with rapamycin or its analogue (Everolimus) attenuates age-related loss of muscle mass and function in rodents [115,116]. Human data reporting the status of AKT/mTORC1 signaling in sarcopenia is limited, due to the paucity of standardised clinical studies. However, early clinical studies suggest that the AKT/mTORC1 pathway may also be mis-regulated in old human muscles [117,118], possibly with low efficiency of AKT signalling [117] and increased basal levels of phosphorylated mTORC1 and its downstream target S6K1 [118], these data being consistent with evidence in rodent sarcopenia.

Thus *loss of proteostasis* is endorsed as a strong hallmark of sarcopenia in rodents, with limited but emerging evidence in humans.

### Inflammation

Low-grade chronic inflammation present during ageing also contributes to disturbed proteostasis and muscle atrophy. As mentioned above, age-related activation of RTEs and genomic instability may trigger an innate immune response, and inflammation. Intrinsic age-related dysregulation of the inflammatory system, and other cells of the innate immune response, affects many cellular events [119–121]. Transcriptomic and proteomic data from human and rodent muscles identify upregulated proinflammatory pathways as major hallmarks of ageing muscle [6,77,78,113,122,123]. Discovery proteomics in human muscles show that age increases expression of nuclear factor kappa-B (NF-κB) activators and decreased expression of NF-κB attenuators [6]. Increased inflammatory signalling may be one of the drivers of disturbed proteostasis in sarcopenia, since pro-inflammatory cytokines activate the NF-κB pathway to induce protein degradation via the ubiquitin–proteasomal pathway in humans and rodents [124] (reviewed in [125]). A study of 71 Brazilian community-dwelling older women (age range 66–96 years), that measured cytokines in blood as biomarkers of inflammation, showed highest blood levels of IL8, sTNFr-1, and sTNFr-2 for the more advanced sarcopenia [126].

Such age-related chronic inflammation is associated with increased ROS that can cause reversable and irreversible oxidation of DNA, proteins and lipids and other cellular components, with adverse effects on many tissues [51,52,127]. As a vicious cycle, damaged organelle macromolecules accumulate in aged cells and contribute to a pro-inflammatory state [120]. Indeed, a classic biomarker of ageing is lipofuscin that can provide a lifetime history of exposure to chronic oxidative stress: lipofuscin is an autofluorescent product of irreversibly oxidised macromolecules that accumulate in post-mitotic cells such as ageing human skeletal muscles [128].

Reactive oxygen species (ROS) are generated in several compartments and organelles within the muscle cells, including mitochondria, sarcoplasmic reticulum, and sarcolemma, and increased levels are strongly associated with inflammation. Under normal physiological conditions, ROS modulate many normal biological processes such as signal transduction, cell proliferation, stimulation of antioxidant systems, and apoptosis [50]. However, high levels of ROS can result in dysregulation of signalling pathways and oxidative damage to mitochondria, proteins, lipids, RNA and DNA in muscle cells, contributing to muscle atrophy and sarcopenia [52,129], with sustained inflammation and increased ROS implicated in many age-related disorders [51].

Increased chronic pro-inflammatory signalling in old muscles, may result from failing mitochondria quality control [130] (see *Hallmark: Mitochondrial dysfunction*) that is strongly linked to many age-related disorders [51], increased pro-inflammatory cells (see *Immunoageing*, part of *Hallmark: Altered intercellular communication*), and atrophic myofibres may also contribute; further studies are required to clarify these mechanisms. Additionally, as discussed above, de-repression of chromatin which results in exposure and activation of retrotransposons may partially be responsible for driving innate immune response and inflammation in old muscles [73,74].

In conclusion, this pro-inflammatory environment is endorsed as a moderate and emerging hallmark of skeletal muscle ageing and potentially sarcopenia in humans, as it may disrupt various aspects of organ homeostasis and function (see also *Immunoageing*), with a complex mis-regulation of many interconnected pathways.

### Deregulated nutrient sensing

A nutrient is a substance used by an organism to grow, survive, and reproduce. They include energy-providing macronutrients (e.g., carbohydrates, proteins, fats) and micronutrients (e.g., vitamins, minerals) required for metabolic and physiological functions. There is a preferential atrophy of fast-twitch glycolytic (type 2X) myofibres with age [31,33,131–135], which accounts for the age-related reduction in human thigh muscle lean cross-sectional area [31,134]. Fast-twitch glycolytic myofibre atrophy also occurs in states of actual or perceived nutrient scarcity (e.g., starvation, cancer, and diabetes mellitus) [132,136] suggesting that deregulated nutrient sensing is an important hallmark of skeletal muscle ageing.

Insulin stimulates glucose uptake into myofibres, and low muscle mass is a risk factor for insulin resistance [137]. Glucose transporter (GLUT) 4 is the main insulin-regulated transporter in adult skeletal muscle [138] regulated by a complex process involving AKT activation (phosphorylation) [139]. Elevated basal mTORC1 activity [115,117] that inhibits AKT activation via a negative feedback loop may contribute to mis-regulated AKT signalling in old muscle. This could help explain the right-shift in insulin-glucose-dose-response curve with age (i.e., a greater insulin concentration is required for the same degree of glucose uptake) [140]. Skeletal muscle is responsible for 80% of postprandial glucose uptake [141], thus a right-shift in this relationship may account for a significant proportion of insulin resistance [142]. In fact, reduced skeletal muscle glucose uptake can precede Type 2 diabetes onset by 10–20 years [143]. However, the relationship between insulin resistance and skeletal muscle is bidirectional: older adults with sarcopenia have an increased risk of developing diabetes, whilst adults with diabetes have an increased risk of sarcopenia [144]. This relationship is emphasised by a study in 72-year-old men and women where reduction of daily activity for 2 weeks impaired insulin sensitivity and reduced leg lean mass [145] -an effect which persisted for two weeks after return to habitual activity [146].

Slow type 1 myofibres mainly use lipid as a fuel. Muscle lipid accumulation is associated with sarcopenia and has been linked with insulin resistance and mitochondrial dysfunction although the exact mechanisms remain unclear. Although some of this lipid accumulation is likely due to inactivity rather than ageing *per se* [147], the preferential oxidation of serum-free fatty acids, instead of intramyocellular lipid as well as larger lipid droplets that are *not associated* with mitochondria, are differences that persist when activity levels are controlled for [148–150]. Highly trained individuals also exhibit increased muscle lipid, but droplets tend to be smaller and *associated* with mitochondria [149]. This suggests the relation of lipid droplets to mitochondria as well as their absolute quantity is important.

As well as insulin resistance and increased lipids, older muscles display anabolic resistance; this term describes reduced protein synthesis in response to amino acids, insulin, and exercise triggers [151–154]. Although basal muscle protein synthesis seems not to be affected by age, response of old muscles to infused amino-acid-glucose mix is blunted [118].

It is possible that at least some of the age-related deficit in response to amino acid infusion is due to impaired vascularisation rather than contractile protein synthesis. When amino acid infusion is combined with sodium nitroprusside to induce local vasodilation, there is no longer an age difference in protein synthesis [155,156]. This implies that reduced skeletal muscle capillary density, and compromised endothelial wall function are two likely contributors [132].

AMP-activated protein kinase (AMPK) is an energy sensor protein that switches cell pathways between anabolism (protein and lipid syntheses) and catabolism (e.g., protein breakdown) in response to the intracellular AMP/ATP ratio or by phosphorylation. In a study of human twins, genetic factors had little effect on AMPK expression [157]. However, the expression the AMPK complex activated during exercise (α2β2γ3) was higher in fast-twitch fibres, higher in men (than women) and reduced with age [157]. This myofibre-type specific reduction with age accords with a rodent study that examined the contraction-evoked effects on AMPK activation in fast- and slow-twitch myofibres [158].

Overall, there is moderate evidence that altered nutrient sensing is a hallmark of skeletal muscle ageing and sarcopenia, linked with diabetes in a bidirectional manner in humans.

### Mitochondrial dysfunction

Mitochondria are derived from early endosymbiotic bacteria. They are the main source of cellular energy, and have many other roles including hormone production, calcium buffering, and generation of ROS [159]. However, under pathological conditions mitochondria may produce excess or altered ROS, damaging cellular components (e.g., proteins, lipids, nucleic acids) and contributing to cellular and systemic inflammatory responses [159–162]. There is also emerging evidence that mitochondria may be key players in cellular senescence and the senescence-associated secretory phenotype (SASP) [163] (see *Hallmark: Cellular senescence*).

Decline in mitochondrial function appears to be a prominent hallmark of loss of skeletal muscle mass and function with ageing and inactivity [164] and is one of the few hallmarks directly linked to functional decline [165,166]. An increase in the number of COX^−^ myofibres with age is a consistent finding in human skeletal muscle and correlates with accumulation of mtDNA mutations [54,167,168] (see *Hallmark: Genomic instability*). Above the age of 40 years, up to five COX^−^ myofibres are considered within normal limits, while under 40 years these indicate pathology [168,169]. However, controversy exists regarding whether the reduced mitochondrial oxidative capacity is explained by reduced mitochondrial volume alone [149,170], or a deficit in oxidative capacity, in addition to reduced mitochondrial number/volume [171,172]. Genome-wide transcriptomic analyses show down-regulation of transcriptional signature of genes linked to mitochondrial proteostasis and function, in sarcopenic muscles of rats [77,113] and humans [122,160]. Sarcopenic individuals of different ethnicities demonstrate a decline in transcriptional signature of mitochondrial function [172]. Notably, transcriptional down-regulation of gene pathways linked with mitochondrial function (tricarboxylic acid (TCA) cycle, oxidative phosphorylation, mitochondrial respiration) have the strongest association with human muscle mass and function [172]. Congruent with gene expression data, discovery proteomics from skeletal muscles from people aged 20–87 years showed an age-related decrease in proteins related to energetic metabolism, including those related to the TCA cycle, mitochondria respiration, and glycolysis [6].

In terms of a link with inflammation, analyses of 669 adults from the Baltimore Longitudinal Study of Ageing showed association between low mitochondrial oxidative capacity and markers of chronic systemic inflammation [160], suggesting that damaged mitochondrial DNA (see *Hallmark: Genomic instability*) and excess production of ROS by dysfunctional mitochondria could trigger inflammation [160]. To this end, levels of circulating cell-free mtDNA were found to be an independent risk factor for sarcopenia [173].

Clearance of damaged and dysfunctional mitochondria via autophagy/mitophagy may be impaired in old skeletal muscles because of a compromised catabolic process [174] that may also contribute to inflammation [120]. Sustained activation of the mTORC1 signalling in sarcopenic skeletal muscles has been suggested as a mechanism that inhibit mitochondrial turnover (clearance) via supressing autophagy [175]. However, while activation of the mTORC1 signalling and benefits of pharmacological inhibition of this pathway in old muscles has been relatively well studied in rodents [115,116], human data are lacking.

Interestingly, healthy old muscles can also carry healthy mitochondria, with mitochondrial content and mitochondrial respiratory function preserved in skeletal muscles of 85+ year old men and women who were healthy, active and did not have a deficit in muscle strength [176]. Therefore, it is not clear if mitochondrial decline causes sarcopenia or is a result of sarcopenia [161]. However, decline in mitochondrial function is certainly a feature of sarcopenic muscle, and inactivity (which increases with age), and is likely a significant contributor to mitochondrial demise, since exercise improves oxidative capacity independent of age [177].

### Cellular senescence

Cellular senescence represents a state of permanent cell cycle arrest in response to various stressors. Senescent cells arise via different senescence programs, including replicative senescence (i.e., telomere shortening), DNA damage-induced senescence, oncogenic stimulation, mitochondrial dysfunction-associated senescence, and oxidative stress-induced senescence [178,179]. Senescent cells accumulate with ageing and disease, in mitotic and post-mitotic human and animal tissues [180,181] hampering their regenerative capacity [123] and promoting disease development. Senescent cells secrete a range of extracellular modulators such as cytokines, chemokines, growth factors, and extracellular matrix degrading proteins termed SASP [182–184], which contribute to systemic dysfunction in ageing and disease [179,183]. Senescent cells are highly heterogenous at the transcriptomic level [123], and their identification and quantification remain difficult because of their scarcity [123] in the absence of a universal senescence marker [179,185]. Nonetheless, the presence of senescent cells has been described in multiple human tissues and implicated in the pathogenesis of several age-related diseases [181]. In animal models, the clearance of senescent cells has been shown to improve the ageing phenotype and alleviate age-related pathologies [186,187].

There is little information for human skeletal muscle *in vivo*, regarding the extent that senescent cells and SASP accumulate in post-mitotic myonuclei of myofibres, satellite cells, and other mitotic interstitial cells, and how they might contribute to muscle ageing and sarcopenia. Some observations in aged murine models with several markers of cellular senescence have been replicated in human studies, whereas others have not (reviewed in [188]). Early reports of bulk muscle tissue showed an increase in mRNA levels of the senescence-associated genes involved in cell cycle arrest/DNA damage (i.e., *p21CIP1* and *p53*) in older women aged 65–71 years [189]. Cultured human myoblasts did not show elevated senescence markers p16INK4a, senescence-associated β-galactosidase (SA-β-Gal) (a marker of lysosomal overload), and DNA damage response marker γH2A.X [190] with ageing as observed in rodent studies [188]. Also, skeletal muscle transcriptome in the GESTALT study of bulk muscle tissue from 53 healthy humans (aged 22–83 years) showed differential expression of mRNA encoding proteins of cellular senescence, but not p16INK4a protein [191], a primary mediator of cell-cycle arrest and a promoter of cellular senescence [192].

Studies using bulk muscle tissue fail to determine the spatial distribution of the signal across different cell-types. This was addressed recently in a comprehensive assessment of young and aged mice and 52 human muscle biopsies (including 22 older adults aged 69.8 years) [193] with spatially resolved methods of single myofibres [194]; and also in a lifecourse study of 40 middle-aged and older adults [195]. qPCR revealed increased *p16INK4a* and *p21CIP1* mRNA expression in older human muscle compared to young, which were negatively associated with muscle oxidative capacity, maximum oxygen consumption, and strength (leg extension) [193]. Immunohistochemistry staining revealed higher frequency of p16INK4a-positive nuclei (within and outside human myofibres) and higher frequency of γH2A.X staining colocalising to telomeres (i.e., telomere-associated DNA damage foci or TAF) [193]. Senescence markers HMGB1 (High Mobility Group Box 1), TAF, and Lamin B1 were associated with muscle mass and function in the lifecourse study and observed to be stronger in women than in men [195]. Taken together, the results revealed the core elements of the senescence programme in aged human muscle decline, with implications for muscle quality and function [193,195].

A combination of single-cell transcriptomics and a senescence-cell enrichment sorting protocol [123] detected a new senescence-cell niche comprised of three main cell populations (satellite cells, fibroadipogenic progenitors, and myeloid cells) in regenerating muscles after experimental injury, for young (3–6 months) and old (>28 months) mice. Also, human muscles *in vivo* with damaged areas (aged 69–85 years) were characterised by upregulated cellular stress pathways (oxidative and metabolic) and downregulated DNA-damage and mitochondrial response pathways implicated in inflammation and fibrosis [123]. These senescent cells and their inflammatory secretome can blunt muscle regeneration *in vivo* after experimental injury in young and old mice [123].

The role of SASP proteins in human muscle ageing was investigated in the LIFE study of over 1,300 older women and men aged 70–89 with sedentary lifestyle and the risk of mobility disability (Short Physical Performance Battery [SPPB] score 7–10). The top 10 SASP proteins in blood (e.g., actin A, VEGFA, IL15, IL6, and MMP7) were inversely associated with disability and the SPPB score and its individual components [196]. These SASP biomarkers had superior predictive power for poor physical performance (SPPB ≤7), slow gait speed (≤0.8 m/s), low muscle strength (grip strength <16 kg [women], <27 kg [men]), compared with models with only age, sex, race, and BMI [196].

In summary, the results of these studies indicate that senescent cells and SASP may contribute to muscle ageing and poor muscle function in older sedentary adults; however, a direct association with sarcopenia is unclear as a basis for senescence to be explored as a possible therapeutic target (reviewed in [197,198]).

### Stem cell exhaustion

The main myogenic stem cells of interest in skeletal muscle are a sub-population of satellite cells. Satellite cells give rise to myoblasts during regeneration, they are a heterogeneous population, and the proposal that some intrinsic decline in satellite cell function contributes to human sarcopenia has attracted much attention, with many controversies (reviewed in [199,200]).

The proposed role for satellite cells during sarcopenia assumes that there is extensive intrinsic myonecrosis and regeneration occurring in normal human muscles throughout life: yet this does not seem to be the case. It is widely recognised that regeneration of normal human muscles is of vital importance in response to myonecrosis that usually results from major accidental damage, surgery, or experimental injury. However, in most human muscles (in the absence of major injury, or excessive exercise), it appears that intrinsic myonecrosis is a rare event during activities of normal daily life, even in response to strenuous exercise (reviewed in [22,201]), as discussed below.

#### Low myonuclei turnover and intrinsic myonecrosis throughout life

Human skeletal muscle is a relatively stable tissue with little turnover of post-mitotic myonuclei because TLs in these myonuclei remains almost constant from birth to 86 years [88]. This conclusion is supported by radioisotope labelling of nuclei in human muscles [202] and comprehensively reviewed in [203,204] (see *Hallmark: Telomere attrition*). Compelling evidence for minimal turnover of myonuclei throughout the lifespan comes from experiments in mice where satellite cells were eliminated from all muscles of young adult mice, with no subsequent impact on manifestation of sarcopenia in very old sedentary mice [205], indicating no requirement for adult myofibre size maintenance [206]. This conclusion implies no regular satellite cell-dependent regeneration in these ageing mice, and thus no intrinsic incidence of myonecrosis.

For human muscles across the lifespan, there does not appear to be histological evidence of intrinsic myonecrosis reported [207], although this may be hard to encounter in small muscle biopsies [208] (Figure 2). Morphological changes in very old muscles, such as small myofibres and central myonuclei, may have been mis-interpreted as resulting from regeneration (with assumed prior myonecrosis); instead, they are more likely to result from other changes during ageing such as atrophy, denervation, and myofibre splitting [201]. Further histological investigations would be useful to provide direct evidence of the incidence of myonecrosis in normal ageing muscles of human and other species and in response to damaging exercise.

#### Capacity for muscle regeneration and satellite cells myogenesis in older adults

Because it is difficult to induce myonecrosis in human muscles *in vivo* even when applying damaging eccentric exercise (reviewed in [22]), a group in Copenhagen established an experimental model to induce myonecrosis/regeneration in humans in response to physiological overload using electrically stimulated eccentric contractions [209]. When they compared the response of muscles from young (20–31 years) and older men (60–73 years) [210], muscle regeneration was similar in both groups; thus these *in vivo* studies emphasise that myonecrosis (and hence the need for regeneration) is rare in healthy ageing human muscle in response to normal daily activities including exercise. This study used heathy older individuals, with only minor deficits in muscle function [210]: thus it is hard to determine whether a similar competent regenerative capacity would be evident in muscles of very old adults with clinically diagnosed sarcopenia [211].

Old human satellite cells retain an excellent intrinsic capacity for myogenesis, which was confirmed by an *in vivo* study that transplanted human muscles (from cadavers) into immunodeficient mice, and there was no measurable difference in regeneration observed across the range of ages up to 78 years [212]. However, it is increasingly recognised that age-related progressive detrimental changes in the extrinsic environment, including altered inflammation and increasing fibrosis, can result in delayed kinetics of regeneration and impaired muscle formation in very old hosts [123,207,213,214].

#### Other roles for satellite cells in muscle ageing

Since a major role for satellite cells in the context of necrosis/regeneration does not appear to be supported for sarcopenia, this raises the issue of other possible roles for satellite cells in sarcopenia; such as satellite cell activation in response to transient contractile muscle damage, without fusion to the myofibre [210]: the consequences of this transient activation of satellite cells are not yet understood (reviewed in [22]). The experiment by Fry et al. [205] suggests that satellite cells might modify the ECM and reduce fibrosis, and there is increasing evidence to support some role for activated (and possibly quiescent) ‘non-fusing’ satellite cells for cross-talk with ECM and other components of muscle tissue [206]. Satellite cells are now known to communicate with myofibres, fibroblasts [206], and vascular endothelial cells [215], and other cellular components of muscle tissue with signalling that can involve NMJ [216], biomechanical interactions with the ECM and cilia on satellite cells [217], and the release of small extracellular vesicles (exosomes) [206]. The contribution of such cross-signalling involving satellite cells, to modulation of muscle homeostasis throughout the lifespan and sarcopenia remains to be determined.

In conclusion, while stem cells are of major importance during development for constructing skeletal muscles, evidence for a key role of myogenic stem cells in maintaining normal muscle mass throughout life is lacking, and stem cell exhaustion does not appear to be a relevant hallmark for sarcopenia.

### Altered intercellular communication

Intercellular communication that includes systemic interconnectivity and important signalling between myofibres and other cellular components is the last of the classic nine hallmarks of ageing. Here we discuss extrinsic (outside the myofibres) factors that impact muscle ageing: *immunoageing, neuronal dysfunction*, e*xtracellular matrix dysfunction*, and *reduced vascular perfusion*. Another vital intrinsic component of myofibres *ionic dyshomeostasis* is also described.

### Immunoageing

Age-related changes in inflammatory cells (inflammageing), and other immune cells are all covered by the term immunoageing. There are many systemic changes in the immune system with ageing with one striking aspect being exacerbation of a pro-inflammatory environment, associated with increased ROS (see *Hallmark: Inflammation*). Adverse effects of immunoageing on skeletal muscles are demonstrated by muscle wasting (atrophy), discussed previously (see *Hallmark: Inflammation*). Atrophy also occurs in response to cancer cachexia, that is increased in old mice (compared with young), and further exacerbated by anti-cancer therapies; which is ameliorated by immunomodulation of old macrophages [218], and improved recovery after disuse atrophy in old mice [219]. Similar adverse effects of immunoageing, and the benefits of immunomodulation, are evident for the kinetics of muscle regeneration after experimental injury in old mice [214,220].

Regeneration of old nerves is also impaired because of defective response of Schwann cells attributed to immunoageing, especially hyperinflammation, since the microenvironment of old uninjured nerves is characterised by chronic macrophage infiltration with elevated expression of cytokines, pro-inflammatory markers [221] and oxidative stress with irreversible oxidation of macromolecules [222]. A comprehensive review emphasises that immunoageing has major adverse effects on many aspects of regeneration after experimental injury of old muscles [223]; although intrinsic necrosis/regeneration in healthy muscles with ageing is not a regular occurrence. While there is strong evidence for immunoageing in animals and human, the evidence for broad immunoageing as a hallmark of human sarcopenia is overall moderate.

### Neural dysfunction

**Figure 4:**
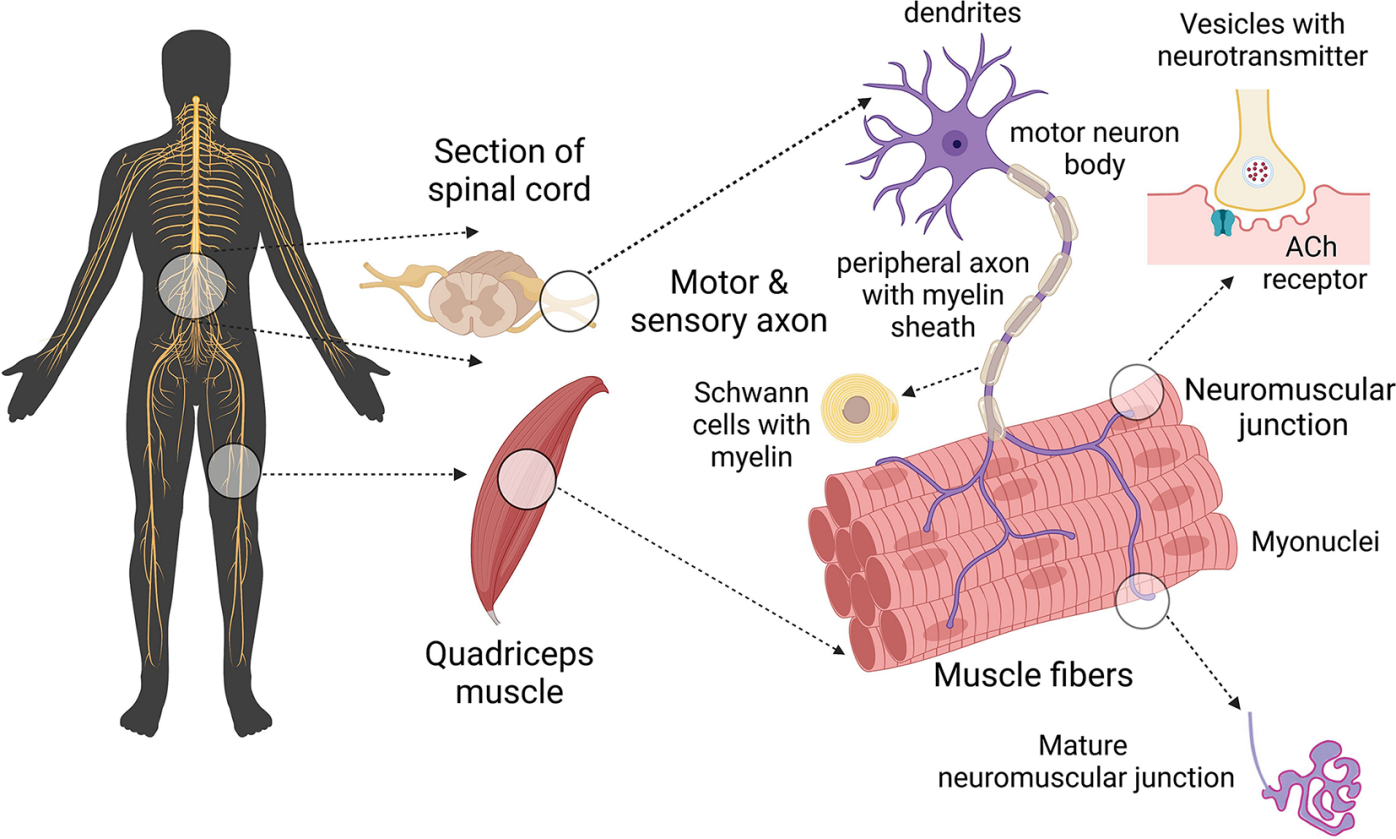
Key components of motor unit and neuromuscular junction Schematic diagram of the motorneuron-skeletal muscle connection that is critical for the transfer of electrical signals for myofibre contraction. The motorneuron cell body in the spinal cord, sends an electrical signal down the motorneuron axon to the NMJ on the surface of an individual myofibre. The pre-synaptic nerve terminal release vesicles containing the neurotransmitter acetylcholine, this binds to receptors on the post-synaptic folds on the myofibre surface (sarcolemma). This signal is transferred along the myofibre surface and then into the T-tubules to initiate contraction. In mature human NMJs, the post-synaptic junction on each myofibre forms complex folds, a typical form of mature NMJ (in red; bottom right). The NMJ is innervated by a single motor axon (in purple; bottom right). Created with BioRender.com. ACh receptor, acetylcholine receptor.

Efficient transmission of electrical signals via the motor neuron-skeletal muscle connection is critical for normal function of skeletal human muscle (Figure 4). There is very strong evidence of age-related changes in neuronal systems associated with loss of function and mass of old human muscles [224,225]. Major age-related changes in skeletal muscles of older humans, including loss of muscle mass (Figure 2), along with shifts in myofibre type were discussed briefly (see ***Human skeletal muscles and ageing*** [22–25,30–38]): the fast-to-slow myofibre transitions during ageing are further supported by proteomic studies [226]. The associated neuronal changes are extensive and include: demyelination of axons caused by oxidative damage to proteins and lipids [227], axonal atrophy, with altered ECM including increased collagen around nerves, loss of motor units, increase in motor unit size, with reduction in motor unit discharge rate, and motor unit remodelling [228,229].

Also well documented are progressive age-related changes in NMJ morphology, including alterations at the pre-synaptic and post-synaptic membranes and terminal Swann cells, with modified transmission of the electric signal through the NMJs [230]. In contrast to rodent studies where there is extensive evidence of NMJ remodelling throughout life, relatively little is known about human NJMs (Figure 4), that are smaller and differ morphologically compared with rodent NMJs. The only comprehensive human study showed no evidence of morphological changes across the lifecourse for NMJs on *peroneus longus* muscles [231], with conflicting observations for other studies on intercostal muscles (discussed in [230]): differences in the extent of sarcopenia between these specific muscles may contribute to such discrepancies. However, there is strong evidence from many human studies that myofibre denervation likely contributes to the decline in physical function in sarcopenia via different mechanisms, including breakdown of the NMJ affecting a single myofibre, or death of a motorneuron leading to denervation or loss of all myofibres in that motor unit [232], or myofibre atrophy contributing to the NMJ breakdown and subsequent denervation. There is selective loss of large fast motorneurons (that innervate fast-twitch myofibres), with re-innervation of these ‘intact denervated’ myofibres by collateral sprouting of slow motorneurons throughout life (reviewed in [225,233]). Such remodelling indicates adaptation and a homeodynamic response to ageing, rather than homeostasis [234].

Confusion can arise when interpreting histological changes evident in normal old human muscles, such as atrophy of fast type 2X myofibres, small diameter myofibres and altered shape of myofibres with central myonuclei, which in other situations are attributed to regenerated muscles, since it seems likely that these may instead result from progressive denervation, atrophy, increasing fibrosis and splitting of myofibres due to remodelling [23,201,207,208,235].

Ageing also adversely affects the function of muscle spindles, small sensory organs within muscles (proprioceptors) that have a marked impact on gait, balance, and frailty [236]. Recent studies show that ageing reduces the sensory excitatory feedback from muscle spindles to motoneurons in the spinal cord, to compromise motorneuron function, with adverse effects on locomotion; demonstrated in mice initially and recently in old primates and humans [237,238]. Exercise has benefits on these age-related changes [239,240].

There is increasing recognition of the benefits of physical exercise on molecular markers of NMJ stability and myofibre denervation (reviewed in [241]) and neural markers such as motor unit discharge rate [242] in healthy older adults. Importantly, physical exercise is one of the few interventions that can prevent and mitigate sarcopenia (reviewed in [243–245]), with the adverse effects of sedentary behaviour emphasised [246].

Despite extensive evidence of neural dysfunction as a hallmark of human sarcopenia, it remains unclear whether sarcopenia is initiated and driven primarily by changes in nerves, or by myofibres, or both [247]. In humans, there is evidence that alteration in peripheral and central motor neuron excitability and changes in conduction velocity precede neuronal loss [248–251], but how these changes relate to simultaneous changes occurring within human muscle has, to our knowledge, not been explored. A lifecourse approach with a particular focus on the age around which muscle mass and different aspect of neuronal function first starts to decline [26] is needed to disentangle the relative contribution of nerves and myofibre to skeletal muscle ageing and sarcopenia.

### Extracellular matrix dysfunction

The ECM that surrounds each myofibre and the whole muscle, is of crucial importance for skeletal muscle function (Figure 1). Basement membrane is specialised laminin-rich ECM in intimate contact with the myofibre surface (sarcolemma) which transfers and integrates the force, generated by contractile proteins within the myofibre, across the sarcolemma and out to strong collagens in the interstitial ECM compartment, to move parts of the body [252]. The ECM also plays major roles in muscle formation and regeneration (reviewed in [253,254]).

Complex changes in ECM gene expression across the lifespan are a feature of skeletal muscle in ageing rodents [113,255], and many differences in ECM composition are described for human and rodent muscles during ageing (reviewed in [256]). Analyses of ECM gene expression in muscles of young and older men (aged 70 years), showed changes in transcriptome of collagen I and some matrix metalloproteases (MMPs) and their inhibitors (involved in ECM remodelling), which were marked with age and in response to exercise [257]. ECM changes in older muscles, nerves and other tissues are widely reported in humans and animals, including higher collagen content and increased oxidation (glycation) and crosslinking, with decreased collagen turnover (reviewed [258]) that contributes to impaired function of old muscles [259]. Consequently, ageing is associated with pronounced interstitial muscle fibrosis and increasing ‘stiffness’ (i.e., reduced elasticity). Such altered stiffness affects biomechanistic, mechanotransduction and function, and can also impair myogenesis [22]. The complexity of changes in many ECM proteins and their turnover in response to different forms of exercise, unloading, and ageing in human muscles is the focus of a recent review [260]. Increased fibrosis also impairs myogenesis and re-innervation of nerves, with major implications for the efficacy of regeneration in old humans and animals [253].

Consistent reports state that the age-related loss of muscle mass alone cannot account for the extent of decline of muscle strength: across the neuromuscular system there is extensive remodelling with age, which involves muscles (with changes in architecture and pennation), fascia, and the central and peripheral nervous systems [256]. The altered architecture of old tendons may also contribute to altered biomechanics of contraction, as shown by a doubling in length of the myotendinous junction region with an increase in proximal tendon length (31%) for old mouse soleus muscles [261]. Further analyses of many muscles for mice aged 6–32 months emphasise that such ECM tendon remodelling and shortened myofibres (rather than myofibre loss) appear to play a role in the loss of both mass and function in sarcopenia [262]. Such observations emphasise the broad impact of age-related ECM remodelling in the context of sarcopenia which needs further investigations in human studies.

### Reduced vascular perfusion

The dense capillary network surrounding skeletal muscle is dynamic-during muscle contraction blood flow can increase up to 100-fold and delivers oxygen and nutrients whilst removing waste products and heat [263]. There is a close anatomical association between capillaries and satellite cells [264], with more capillaries and satellite cells in slow type 1 myofibres (more reliant on oxidative metabolism) compared with fast type 2 myofibres [265]. Studies in young and old men (aged about 24 and 67 years) show that in old muscles the satellite cells of type 2 (fast) myofibres were located at a greater distance from the nearest capillary, and that a single bout of exercise increased the extent of capillarisation, decreased the distance between capillaries and satellite cells and increased satellite cell activation; demonstrating some of the many benefits of exercise on old muscles [266]. Increased skeletal muscle capillarisation is associated with increased muscle mass [267] and improved insulin sensitivity in men [268]. Despite the increased muscle mass, increased skeletal muscle capillarisation was not associated with greater post-absorptive muscle protein synthesis in older men [267]. However, increased skeletal muscle capillarisation is associated with a faster walking speed and less difficulty with daily activities in men and women [269]. Interestingly, sarcopenic subjects have reduced capillary contacts and a reduced capillary to myofibre ratio, with some evidence that the reduction in myofibre capillary contacts occurs prior to or simultaneously with (but not after) loss of muscle mass [270]. The lower capillary density in type 2 myofibres probably contributes to their increased vulnerability to age-related microvascular dysfunction [271].

In summary, human studies demonstrate that reduced myofibre vascular perfusion is an important hallmark of age-related decline in skeletal muscles, and potentially sarcopenia.

### Ionic dyshomeostasis

Skeletal muscle contraction and ionic homeostasis are the major energy expenditures for myofibres, with each accounting for approximately 50% of ATP use [272]. Dysregulated calcium (Ca^2+^) homeostasis is one of the hallmarks of neuronal ageing [273] and there is evidence from several of the epigenetic (see *Hallmark: Epigenetic alterations*) and other physiological studies that it plays a role in skeletal muscle functional decline with age [274,275].

Skeletal muscle contraction and relaxation relies on massive Ca^2+^ efflux from the sarcoplasmic reticulum (SR) via ryanodine receptors (RyR1) and subsequent reuptake of Ca^2+^ into the SR by the SR Ca^2+^ pump. With age, RyR1 becomes progressively oxidated and nitrosylated causing Ca^2+^ leak and consequent SR Ca2+ store depletion and muscle weakness [274–276]. Mitochondria are tethered to the SR in the region of RyR1 [277] and SR Ca^2+^ efflux via RyR1 triggers mitochondrial ATP synthesis [278]. However, chronic RyR1 Ca^2+^ leak causes mitochondrial overload and dysfunction as evidenced by the cores (regions lacking mitochondria) that characterise RyR1 myopathies in humans. Although studies in human muscle are limited, in mouse models increased mitochondrial ROS production accelerates RyR1 Ca^2+^ leak [279], and RyR1 mutation causing Ca^2+^ leak impairs oxidative phosphorylation [280] -showing further evidence of the bidirectional link between RyR1 and mitochondria.

Skeletal muscle is critical for whole body ionic homeostasis. Extracellular K^+^ makes up approximately 2% of the total body K^+^ store [281] and of the 98% of K^+^ that is intracellular, approximately 80% is in skeletal muscle [281]. Maintenance of this transmembrane K^+^ gradient is performed by activity of the Na^+^/K^+^ pump and accounts for approximately 7% of skeletal muscle ATP use [282]. Maintenance of the Na^+^/K^+^ transmembrane gradient is critical for the initiation and propagation of an action potential.

However, there is evidence in humans that intramuscular Na^+^ increases with age [282–284] whilst intramuscular K^+^ decreases [284] thus reducing the transmembrane Na^+^/K^+^ gradient. A reduction of the transmembrane Na^+^/K^+^ gradient would be expected to depolarise skeletal muscle resting membrane potential and reduce excitability (the ability to generate and propagate action potentials). There is evidence of depolarisation of skeletal muscle resting membrane potential with age in humans [285,286] as well as reduced skeletal muscle excitability with age in rodents and humans [286–289].

As well as being central to muscle excitability, the transmembrane sodium gradient is important for a wide number of co-transporters [290,291]. The impact on molecules that rely on the Na^+^ transmembrane gradient for co-transport (e.g., amino acid transporters or insulin-independent glucose transporters) has, to our knowledge, not been directly explored. However, several studies from independent groups have found a greater accumulation of H^+^ and inorganic phosphate ions in older skeletal muscle from men and women following exercise [292–295]. This was not due to a deficit in ATP production [293] nor a deficit in perfusion [292]. Therefore, the mechanism of this proton and inorganic phosphate accumulation is not known. We speculate that it may be of relevance that two out of the three transporters that can extrude H^+^ from skeletal muscle are sodium dependent and bicarbonate import relies on the sodium gradient [296]. The wider impact of alterations in ionic homeostasis remains to be determined but is worth pursuing as a potentially tractable mechanism contributing to sarcopenia.

### Clinical relevance and translational potential

**Figure 5:**
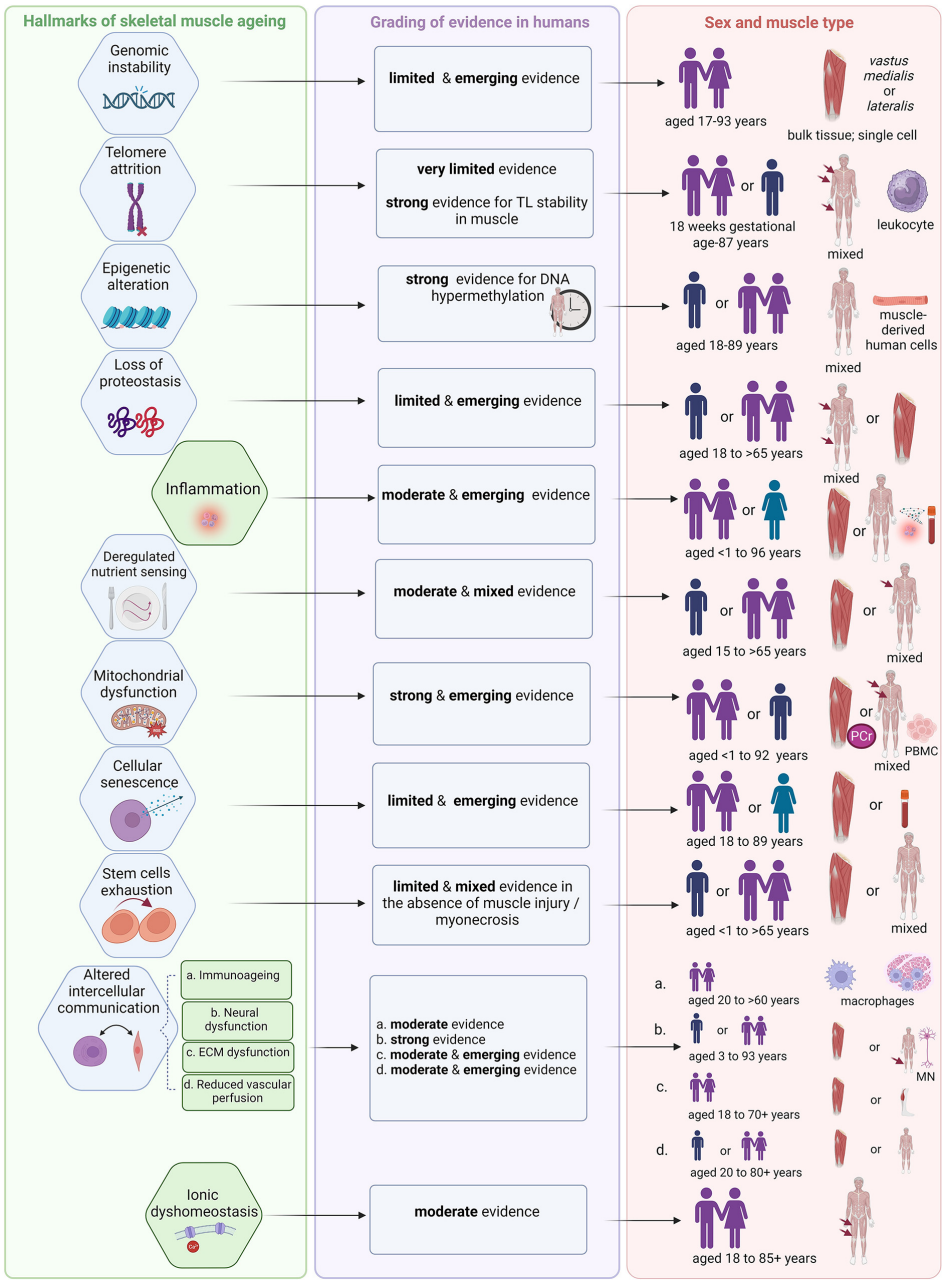
Summary of evidence from human studies for each hallmark of ageing applied to skeletal muscle For each of the nine classic (blue) and five novel hallmarks of ageing (green), the strength of evidence for human skeletal muscle ageing is graded in the middle column and depicted in the end column according to the sex (with age range) and type of muscle (*vastus lateralis* or mixed) most used in these studies. While specific data are available and emerging for some of these hallmarks in the context of sarcopenia (see individual *Hallmark* sections) these data are limited. Because of the relative paucity of studies for women and for investigating sex-specific differences across the lifespan for hallmarks of ageing, possible sex-related differences could not be determined at this time. For example, out of six individual studies included for *Hallmark: Cellular senescence*, only one feasibility pilot study explored senescence markers in men and women separately [195]. Moreover, consensus on clinical diagnosis of sarcopenia is still emerging: consequently, earlier studies likely included sarcopenic individuals who were not diagnosed as such. Therefore, at this stage, it is appropriate to evaluate the hallmarks based on evidence for normal ageing human muscle. This provides the background for progression of sarcopenia (as an adverse manifestation of normal ageing) [10,11] and, in limited cases, provides direct evidence of involvement of a hallmark in sarcopenia. Created with BioRender.com. ECM, extracellular matrix; MN, motor neuron; PBMC, peripheral blood mononuclear cells; PCr, phosphocreatine by ^31^P magnetic resonance spectroscopy; TL, telomere length.

The clinical relevance and translational potential of the proposed hallmarks of ageing skeletal muscle can only be realised by the field within a context of understanding the findings from human and animal studies to date. This review has sought to enable this through describing the nine classic hallmarks of ageing as first described by Lopez-Ortin et al. [1] and five novel hallmarks in the context of human skeletal muscle ageing and sarcopenia (Figure 3). We summarise the likely relevance of these hallmarks for normal ageing human skeletal muscle in Figure 5. This information is combined with text that comments on the implications for sarcopenia, based on early studies that did not differentiate between healthy ageing and sarcopenia, and more recent studies that made these distinctions based on clinical definitions of sarcopenia [10,11].

As indicated in Figure 5, the body of evidence to support *loss of proteostasis, deregulated nutrient sensing*, and *mitochondrial dysfunction* as major hallmarks of skeletal muscle ageing, is large and persuasive in animals, and limited, moderate and strong, respectively in humans. There is overall moderate evidence for the role of altered *intercellular communication* in skeletal muscle ageing in humans that, because of its complexity covering factors extrinsic to myofibres, we subdivided into *immunoageing* (includes *inflammation*), *neural dysfunction, extracellular matrix dysfunction* and *reduced vascular perfusion*. Evidence for the role of *extracellular matrix dysfunction* and *reduced vascular perfusion* is moderate but remain largely underexplored in individuals with diagnosed sarcopenia. *Neural dysfunction* has a strong role in human muscle ageing with some evidence that resilience to sarcopenia has a neural basis [228]: however, better understanding of changes in women is needed (Figure 5). *Ionic dyshomeostasis* is a novel hallmark, with moderate and emerging evidence from human studies of its involvement in skeletal muscle ageing but no evidence of its role in sarcopenia. The evidence for *genomic instability* and *cellular senescence* was limited but, especially in the latter case, has gained attention. Evidence for DNA hypermethylation (*epigenetic alteration*) in ageing human muscle was strong. However, the evidence for *telomere attrition* and *stem cell exhaustion* as key hallmarks of ageing in skeletal muscle is limited and weak.

This review includes a large body of literature investigating the role of the hallmarks of ageing in skeletal muscle and we make four key observations which potentially limit the clinical and translational potential. Firstly, although there is an abundance of superb scientific study in various animal models, there is a relative paucity of work confirming its translation into humans -this needs to be a priority. Secondly, there is a lack of diversity in the human participants of research studies for example characterised by under-representation of women. Thus, sex differences in the hallmarks of ageing in skeletal muscle and their role in the development of sarcopenia in men and women are outstanding questions and a priority for future work. Thirdly, few studies use a lifecourse approach involving participants across all ages with most human studies simply comparing young and old. To identify mechanisms and develop therapies for sarcopenia, the lifecourse approach adds a unique value for advancing translational research [42] (reviewed in [297]).

#### Impact of exercise and nutrition

Despite the intense research into molecular targets and potential drug candidates for sarcopenia using *in vivo* and *in vitro* models [298], the only intervention in human studies that has shown translational potential for sarcopenia [299], by possibly attenuating several hallmarks of ageing, is exercise [300–303]. Many reviews support the pleiotropic and system-wide benefits of regular exercise (both aerobic and resistance), with the potential to counteract age-related diseases, including sarcopenia, by ameliorating hallmarks of ageing [300–303]. Although the relationship between exercise and the hallmarks of skeletal muscle ageing is outside the scope of this review, several pertinent observations are highlighted here. These include studies showing malleability of telomeres in myonuclei due to exercise (i.e., endurance exercise and strength training) [89,90], benefits of exercise on molecular markers of NMJ stability and myofibre denervation (i.e., life-long endurance and resistance exercise) [241], ionic homeostasis (i.e., isometric contractions exercise) [292–295], favourable ECM gene expression involved in ECM remodelling (different resistance exercise modalities) [257], protein turnover [260] and capillarisation, and satellite cell activation in response to exercise in older muscles (i.e., resistance exercise, sedentary versus physically active lifestyle) [265]. Although these studies varied by the type of exercise, duration and intensity of exercise programmes, and the terms used to define self-reported physical activity (thus preventing any meaningful in-depth evaluation of their differential effects on the hallmarks of ageing), they all endorse translational benefits of exercise for better muscle ageing. Exercise has the potential to target multiple hallmarks of skeletal muscle ageing and is the only intervention shown to prevent and mitigate sarcopenia in humans (reviewed in [243–245]) [299].

Evidence from human randomised controlled trials has been accumulating for the beneficial effects of nutritional supplements such as whey protein (with or without vitamin D) [304] and omega-3 fatty acid supplementations [305] in muscle function in older adults with frailty and sarcopenia. There is a vast literature investigating various combined interventions of exercise and nutritional supplements and future work needs to determine how these might ameliorate hallmarks of ageing [306] in the context of skeletal muscle. Another promising area or research related to nutrition includes the gut and microbiome dysbiosis [2] during skeletal muscle ageing [307]. Valuable studies in mice show that changes in the gut microbiome via bacteria depletion, faecal transplantation, and various supplements (e.g., probiotics, prebiotics, and short-chain fatty acids) directly affect muscle phenotypes. Mechanistic studies in mice reveal that these treatments may ameliorate different cellular mechanisms and hallmarks of ageing (e.g., mitochondrial dysfunction, lipid oxidation, inflammation, and muscle fatigue). However, human data related to the role of microbiome and skeletal muscle mass and function have been inconsistent [307] and little is known yet about how the gut microbiome may regulate muscle with ageing.

#### Intercellular connectivity

Although each hallmark is distinct, they are intricately linked and often influence each other creating a network of interactions that drive the ageing process (Figure 3). The interconnectedness, integration, and entanglement of hallmarks of ageing has been discussed in detail in López-Otín et al. landmark papers [1,2]. Recent evidence about causes of mammalian ageing using a system termed ‘ICE’ (inducible changes to the epigenome) in animal models revealed that the cellular response to double-strand DNA breaks erode the epigenetic landscape and advance ageing via other mechanisms (hallmarks), including senescence and DNA methylation [308]. Here we briefly highlight the following hallmark interconnectedness relevant for skeletal muscle ageing. For example, genomic instability can lead to mitochondrial dysfunction which impairs oxidative phosphorylation, impacting the ability to maintain ionic homeostasis and, therefore, neural signalling. Circulating mtDNA released by damaged mitochondria may drive chronic inflammation. Such inflammation is exacerbated by senescent cells that secrete pro-inflammatory factors-triggered by cytosolic mtDNA [163] -further driving senescence and oxidative stress. Also of increasing interest is wider interconnectivity between skeletal muscles and the brain via circulating systemic factors produced by contracting muscles, with links between sarcopenia and many aspects of cognitive dysfunction [166,309,310]. These diverse examples emphasize the interconnectedness of the hallmarks of ageing muscle and sarcopenia, and the complexity and multifaceted nature of the ageing process.

#### Consensus definition of sarcopenia

Progress in clinical and translational research for sarcopenia depends not only on synthesising and recognising the interconnectedness of cellular and molecular findings from representative human studies across the lifecourse but requires an agreed consensus definition of sarcopenia [311]. Recent meta-analysis of observational studies revealed seven definitions and diagnostic criteria for sarcopenia, the European Working Group on Sarcopenia in Older People (EWGSOP) being one of them [312]; and the Global Leadership Initiative in Sarcopenia (GLIS) aims to develop a single clear definition of sarcopenia that can be utilised worldwide [12]. A single clinical definition of sarcopenia could facilitate human studies that employ a lifecourse approach and involve diverse participants. Such studies, focussed on the most promising hallmarks with strongest human evidence (Figure 5) will be key to realise the clinical relevance and translational potential of hallmarks research linked to ageing skeletal muscle and sarcopenia.

## Conclusions

This review provides a broad overview of the importance of hallmarks of ageing to the specific situation of human skeletal muscle and their implications for sarcopenia. We conclude that there is strong evidence for *epigenetic alteration, mitochondrial dysfunction, neural dysfunction*, and moderate evidence for *inflammation, deregulated nutrient sensing, immunoaging, ECM dysfunction*, and *reduced vascular perfusion* as hallmarks for skeletal muscle ageing, with their relevance for sarcopenia evolving.

We recognise that many human studies use only one muscle source (*vastus lateralis*), and we highlight the low representation of older women. We discuss the interconnectedness of these hallmarks mirrored in the complexity of skeletal muscle ageing and pathophysiology of sarcopenia. This intricate linkage provides the opportunity to identify their translational potential by targeting several hallmarks simultaneously with anti-ageing interventions, with exercise as an exemplar.

## Data Availability

This is a narrative review and not an original research paper.

## Open Access

Open access for this article was enabled by the participation of Newcastle University in an all-inclusive *Read & Publish* agreement with Portland Press and the Biochemical Society under a transformative agreement with JISC.

## Abbreviations

Ach: acetylcholine
AKT: AKT-protein kinase B
AMPK: AMP-activated protein kinase
ATP: adenosine triphosphate
BMI: body mass index
Ca^2+^: calcium ion
COX: cytochrome *c* oxidase-negative
CpG: 5′-C[cytosine]-phosphate-G[quinine]-3′
dmCpG: differentially methylated CpG
DMR: differentially methylated regions
ECM: extracellular matrix
ETS: electron transport system
EWAS: epigenome-wide association study
EWGSOP: The European Working Group on Sarcopenia in Older People
GLIS: The Global Leadership Initiative in Sarcopenia
GLUT1: Glucose transporter 1
GLUT4: Glucose transporter 4
GTEx: The Genotype-Tissue Expression project
H^+^: hydrogen ion
HDMC: muscle-derived human primary cell
*HOX*: homeotic genes
HSS: The Hertfordshire Sarcopenia Study
ICE: inducible changes to the epigenome
IFN-1: type-1 interferon
IGF-1: insulin-like growth factor 1
IL15: interleukin 15
IL6: interleukin 6
IL8: interleukin 8
IMCL: intramyocellular lipids
K^+^: potassium ion
LINE-1: long-interspersed element 1
MAPK: mitogen-activated protein kinase
MMP7: matrix metalloproteinase-7
MMP: matrix metalloproteinase
MRI: magnetic resonance imaging
mtDNA: mitochondrial DNA
mTOR: mammalian target of rapamycin
mTORC1: mammalian target of rapamycin complex 1
MYH1: myosin heavy chain isoform 1
MYH2: myosin heavy chain isoform 2
MYH7: myosin heavy chain isoform 7
Na^+^: sodium ion
NF-κB: nuclear factor kappa-light-chain-enhancer of activated B cells
NMJ: neuromuscular junction
OXPHOS: oxidative phosphorylation
PCR: polymerase chain reaction
PI3K: phosphatidylinositol 3 kinase
qPCR: quantitative polymerase chain reaction
ROS: reactive oxygen species
RTE: retrotransposable element
RyR1: ryanodine receptor 1
S6K1: ribosomal protein S6 kinase β-1
SA-β-Gal: senescence-associated β-galactosidase
SASP: senescence-associated secretory phenotype
SPPB: short physical performance battery
SR: sarcoplasmic reticulum
sTNFr1: soluble tumor necrosis factor receptor-1
sTNFr2: soluble tumor necrosis factor receptor-2
T-tubules: transverse tubules
TAF: telomere-associated DNA damage foci
TCA: tricarboxylic acid
TLs: telomere lengths
VEGFA: vascular endothelial growth factor A
